# A novel approach for predicting risk of vector-borne disease establishment in marginal temperate environments under climate change: West Nile virus in the UK

**DOI:** 10.1098/rsif.2021.0049

**Published:** 2021-05-26

**Authors:** David A. Ewing, Bethan V. Purse, Christina A. Cobbold, Steven M. White

**Affiliations:** ^1^ UK Centre for Ecology and Hydrology, Benson Lane, Wallingford, Oxfordshire, UK; ^2^ School of Mathematics and Statistics, University of Glasgow, Glasgow, UK; ^3^ Biomathematics and Statistics Scotland, James Clerk Maxwell Building, The King’s Buildings, University of Edinburgh, Edinburgh, UK; ^4^ Boyd Orr Centre for Population and Ecosystem Health, University of Glasgow, Glasgow, UK

**Keywords:** vector-borne diseases, West Nile virus, climate change, mosquito, mathematical model, delay-differential equations

## Abstract

Vector-borne diseases (VBDs), such as dengue, Zika, West Nile virus (WNV) and tick-borne encephalitis, account for substantial human morbidity worldwide and have expanded their range into temperate regions in recent decades. Climate change has been proposed as a likely driver of past and future expansion, however, the complex ecology of host and vector populations and their interactions with each other, environmental variables and land-use changes makes understanding the likely impacts of climate change on VBDs challenging. We present an environmentally driven, stage-structured, host–vector mathematical modelling framework to address this challenge. We apply our framework to predict the risk of WNV outbreaks in current and future UK climates. WNV is a mosquito-borne arbovirus which has expanded its range in mainland Europe in recent years. We predict that, while risks will remain low in the coming two to three decades, the risk of WNV outbreaks in the UK will increase with projected temperature rises and outbreaks appear plausible in the latter half of this century. This risk will increase substantially if increased temperatures lead to increases in the length of the mosquito biting season or if European strains show higher replication at lower temperatures than North American strains.

## 1. Background

The global human disease burden attributed to vector-borne diseases (VBDs) increased drastically in the latter half of the twentieth century [1]. In 2017, VBDs were estimated to account for 17% of human disease burden, an increase of 2.4% since 1990 [2,3], though this is likely to be an underestimate due to gross under-reporting in many endemic countries [4]. This increase has involved diverse vector-borne pathogens and included arrival and establishment in new areas (e.g. dengue, Zika, West Nile virus (WNV), chikungunya) as well as increased incidence and impacts in endemic areas (e.g. Lyme disease, tick-borne encephalitis) [5–7]. Numerous interacting social, ecological and environmental factors have been implicated in recent expansion and outbreaks of these diseases [8]. For example, rising temperatures affect the biting, survival and reproductive rates of vectors as well as the development and survival of pathogens. Furthermore, changing precipitation patterns impact breeding sites in a diverse way for a range of vectors. These climatic effects interact with non-climatic drivers, namely globalization and urbanization, sociodemographics and public health systems [8,9] to shape transmission and spread of VBDs.

Disentangling the impacts of climatic and non-climatic drivers on expansion of VBDs is challenging, creating difficulties in forecasting potential effects of climate change [8,10]. For example, increased incidence of tick-borne encephalitis (TBE) and Lyme disease in Europe and North America is believed to be associated with climate warming expanding the geographical range and lengthening the active season of tick populations, though the complex ecology and epidemiology have made it difficult to implicate climate change as a main driver [6,7]. Likewise, socio-political changes alongside climate change explained increases in TBE in the Baltics in the late twentieth century [11]. Furthermore, *Aedes albopictus*, a vector of dengue, chikungunya and Zika, established in Italy in 1990 and has since spread across much of the Mediterranean basin, with rising temperatures, trade and travel implicated in its introduction and subsequent dispersal [12]. Historically, a lack of surveillance data has limited the attribution of shifts in VBD incidence to climate change [12]. However, direct evidence of climate impacts on VBD incidence has increased for diseases such as dengue and malaria [13] and establishment of VBDs in previously unaffected areas has highlighted the importance of forecasting potential changes in disease distribution and improving preparedness to deal with emerging epidemics [14].

A recent review by Sadeghieh *et al.* [15] found that current approaches to understanding and predicting VBD risk are typically focused on predicting risk in existing endemic zones (88% of VBD models in 1996–2016) rather than forecasting transmission risk in new regions. This disparity is likely because the ecology and epidemiology of disease systems may be poorly understood for epidemic zones. Typically, many studies of vector or pathogen distributions use correlative approaches to link environmental data to species records to describe their environmental niche, allowing inferences to be made on range limits and habitat suitability [16]. Such approaches have been used to link historical temperature anomalies with rates of human WNV incidence in order to predict future WNV distribution across Europe [9]. However, these approaches do not capture the myriad of climate impacts on vectors, hosts and pathogen seasonality which interact to shape patterns of VBD transmission [17]. Novel approaches to predict how these complex and interrelated processes may drive establishment of VBDs in marginal environments outwith their current environmental niche are required. Mathematical models are a flexible approach by which disease risk in marginal temperate environments can be predicted because they directly incorporate fundamental biological mechanisms, enabling predictions regarding the relative impact of climate change to be made for novel combinations of environmental conditions outside those seen in endemic zones [18,19]. In doing so, we can investigate the likely impacts of predicted scenarios, such as the expected increase in the length of vector biting seasons with increasing temperatures [10].

Despite their wide applicability, existing mathematical models of VBDs generally make simplifying assumptions regarding vector or pathogen dynamics, as summarized by Reiner *et al.* [20] in a review of VBD models from 1970 to 2010. The full vector life cycle, which is typically composed of multiple life stages and which impacts disease risk through seasonally varying vector–host ratios and biting rates, is rarely modelled explicitly (included in only 12% of models [20]). This is despite the fact that seasonality in vector, host and pathogen dynamics are strong drivers of VBD cases [21]. Likewise, only 6% of models included temperature effects on the latency period of the pathogen within the vector, while 5% considered temperature effects on adult mortality or biting rates (none of the models which did so studied WNV). Recent studies have highlighted that predictions of VBD risk can be greatly influenced by these assumptions. Specifically, Vogels *et al.* [22] estimated that *R*_0_ values for WNV in Europe could change by a factor of approximately 6 across the range of predicted vector–host ratios. Similarly, temperature-dependence of vector biting and mortality, and pathogen latency, resulted in an approximate threefold increase in *R*_0_ values of WNV across a temperature gradient of 18–28°C. Given this, there is a clear need for modelling approaches which capture the seasonal nature of drivers of VBDs.

We propose a novel approach that explicitly models temperature effects on the timing and seasonal coincidence of events in the pathogen, host and vector life cycle. In doing so, we aim to improve prediction of the risk of VBD establishment in marginal temperate environments under climate change. Using delay-differential equations (DDEs) with environmentally driven delays we incorporate realistic, climate-dependent representations of vector vital rates and pathogen latency, thus capturing the impacts of climate on seasonal variations in vector–host ratios on transmission risk. We apply our modelling approach to the prediction of establishment risk of WNV in the UK, as WNV is a VBD that exhibits high seasonality and which is currently expanding its distribution into Northern Europe, having recently been reported in both Germany and the Netherlands [17,23,24]. WNV is a *flavivirus* primarily transmitted in a cycle between *Culex pipiens* mosquitoes and birds [25] that can spillover to human and equine populations causing encephalitis and death in vulnerable groups [26]. As WNV continues to expand northwards there is growing concern that migratory birds travelling from endemic areas could introduce the virus to the UK [27,28]. However, questions remain around the current and future suitability of the UK climate for WNV establishment. Firstly, are projected temperatures high enough to sustain transmission cycles? If so, will introduction of the pathogen coincide with a period of sufficiently high vector activity? Is the vector biting season long enough to allow sufficient amplification of the pathogen in the vector and host populations to facilitate spillover into humans? Finally, how might likely shifts in the timing of these events affect outbreak risk under future climate scenarios?

## 2. Methods

**Figure 1.**
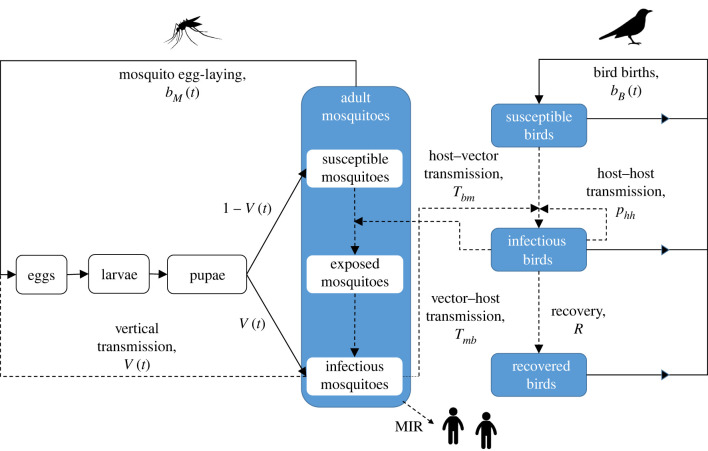
Flowchart showing the relationships between the mosquito, bird and human populations, as defined in the model, and highlighting the processes by which individuals transition between infection classes, subject to the disease-related parameters. All stages have an associated death rate, which is not displayed here for clarity. All disease transmission processes are shown by dashed lines, while life cycle processes are shown by solid lines. Many of the processes are temperature-dependent.

We developed a susceptible–exposed–infectious–recovered (SEIR) vector–host mathematical model with compartments for each life stage of the vector population and for each possible infection status of hosts and adult vectors (figure 1). The model builds upon a previous model of *Cx. pipiens* seasonal dynamics [29], which has been validated against UK field data [30]. It is based on a series of DDEs with temperature-dependent delays to provide a realistic representation of vector seasonality. Temperature-dependence of vector vital rates and pathogen development rates was parameterized from existing laboratory data (see electronic supplementary material, S1–2, [29,30]). Adult vectors are categorized as susceptible, exposed or infectious according to their interactions with an avian host population which experiences seasonal forcing through a varying birth rate, which restricts births to occur in spring and summer, and within which individuals are classified as susceptible, infectious or recovered. Disease transmission between the vector and host populations occurs (following the introduction of a small number of infectious birds) through infectious mosquitoes feeding on susceptible birds and susceptible mosquitoes feeding on infectious birds [25]. Disease transmission within the host population can also occur through host to host transmission and in the vector population through vertical transmission [25]. For simplicity, we do not explicitly model the human population, which are dead end hosts [25]. Instead, we use the minimum infection rate (MIR), which is the number of infectious mosquitoes per 1000 adult females and is a widely used metric to infer the relative risk of human infection by WNV [31].

The explicit temperature-dependence in vector development and survival rates and pathogen replication rates enables predictions of whether projected temperatures are sufficiently high to sustain transmission cycles. By coupling the seasonal vector model with a seasonal host model, we can control the timing of pathogen introduction and make predictions about how timing of the pathogen introduction will affect outbreak risk. The use of temperature-dependent, stage-structured DDEs allows us to capture developmental lags in both the vector population dynamics and the transition of exposed to infectious mosquitoes, facilitating understanding of whether the time between pathogen introduction and the cessation of the active vector season is sufficient to allow amplification of the pathogen to levels which may cause spillover into humans. The flexibility of our mechanistic modelling framework, which is based upon the fundamental biological processes, means that it is straightforward to change parameters, such as the season start and end timings, to understand how risk may change under future climate. While this model is applied to WNV in this case, the underlying mechanistic model framework could readily be adapted to study other pathogens.

We simulated the WNV model under various warming scenarios provided as part of the UK Climate Projections 2018 (UKCP18) report produced by the Met Office [32]. We used the regional projections, which are available on a 12 km grid over Europe and are downscaled from the global projections from the Hadley Centre model (HadREM3-GA705). These projections consist of 12 sets of minimum and maximum predicted daily temperatures (numbered 1, 4, …, 13, 15 as runs 2, 3 and 14 are not provided by the Met Office) across the UK from 1980 to 2080. The other available scenarios are less well resolved and thus would fail to properly drive the model, giving spurious predictions [29] (see electronic supplementary material, S3 for more detail). By using minimum and maximum daily temperatures, we incorporate potential effects of diurnal temperature variation, which has been shown to have large effects on ectotherm vital rates and population dynamics and vector competence, into our model predictions [33]. The WNV model was simulated at this 12 km resolution, with the input temperatures for each WNV model run corresponding to a different independent run of the stochastic UKCP18 climate model, under the assumption that movement between grid squares was negligible as the mean flight range of *Cx. pipiens* is substantially less than 12 km [34]. Each simulation was run for 3 years, with the first 2 years discarded as ‘burn-in’ and the virus introduced in the third year with WNV risk calculated for this third year. For example, to determine the risk in 2079 simulations were started at the beginning of 2077 and run until the end of 2079 with WNV introduction in 2079. This ‘burn-in’ period was included as it was found by simulation that 2 years was sufficient to ensure that the results were not unduly influenced by the initial conditions of the model e.g. initial mosquito population size and composition, initial temperature conditions. The 12 temperature datasets are only provided under representative concentration pathway 8.5 (RCP8.5) which the UKCP18 overview report describes as ‘a world in which global greenhouse gas emissions continue to rise… where the nations of the world choose not to switch to a low-carbon future and so can reasonably be considered to represent a worst-case scenario (see electronic supplementary material, S3 for more detail).

Risk maps showing MIR for each grid square for each of the 12 sets of predicted temperature data were produced for the last year of each decade from 2019 until 2079. Under our model, the vector seasonal abundance patterns and pathogen replication rates will vary temporally across years and spatially across the UK as a result of differences in input temperature and photoperiod (the number of daylight hours per day). Three different times of WNV introduction times were considered: introduction at the end of March, the end of April and the end of May. These timings are consistent with the arrival times of a range of potentially competent migratory hosts such as the swallow, chiffchaff and willow warbler [35], among others as listed by Bessell *et al.* [27]. We also study the potential effects of a lengthening of the mosquito season with increased temperatures, as diapause initiation is known to vary due to both temperature and photoperiod [36]. To understand what effect this might have on WNV risk, we explored reducing the threshold for which 50% of adult females entered diapause to 14 h, corresponding to diapause initiation centred approximately two weeks later at the end of August (dependent on the latitude, which affects daylength such that northern and southern populations will enter and leave diapause at slightly different times).

**Figure 2.**
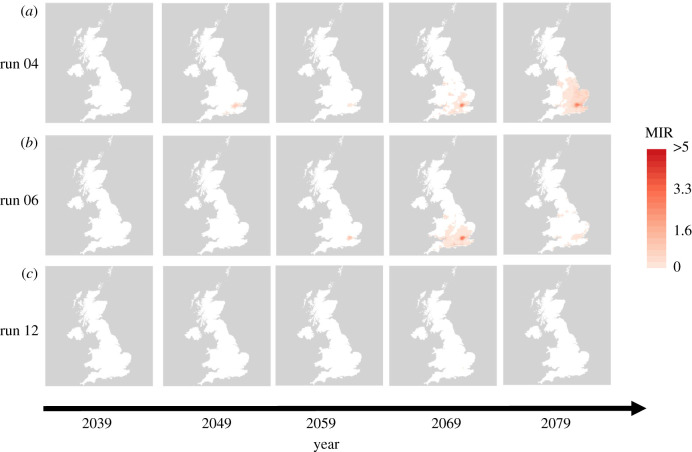
Risk of WNV introduction via late May migrants: risk of WNV outbreak via the arrival of migratory birds at the end of May for the temperatures simulated using UKCP18 model runs 04 (*a*), 06 (*b*) and 12 (*c*). The five plots correspond to results from 2039, 2049, 2059, 2069 and 2079 from left to right. Due to very low estimated risk only maps from 2039 onwards are shown.

**Figure 3.**
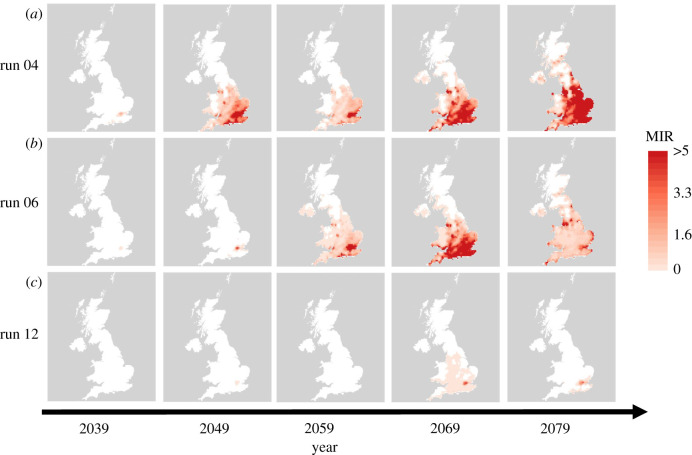
Upper limit of WNV risk given lower thermal minimum for WNV replication: This figure shows the risk of WNV outbreaks via the arrival of migratory birds at the end of May for the temperatures simulated using UKCP18 model runs 04 (*a*), 06 (*b*) and 12 (*c*). The five columns correspond to results from 2039, 2049, 2059, 2069 and 2079 from left to right. In this case, the thermal minimum for WNV transmission has been set to the lower limit of the estimated 95% CI for the parameter, which is 7.3°C [37]. Due to very low estimated risk only maps from 2039 onwards are shown.

Details of the mathematical model used to simulate vector and host population dynamics and on the assumptions regarding WNV transmission between the mosquito and bird populations are given in full in electronic supplementary material, S1. The following further information is given in electronic supplementary material: parameterization of the extrinsic incubation period/viral replication rate (S2.1) [37], parameterization of the WNV transmission processes (S2.2), tuning of vector–host ratios (S2.3), the process by which WNV is introduced (S2.4), the conversion of air temperatures to water temperatures (S2.5), the model history and initial conditions (S2.6) and a table of the parameter values required for the vector life cycle model (table S2). All model code is available on GitHub [38] and is run using the DDE solver of Thompson *et al.* [39]. In the subsequent sections, the maps are presented such that white areas correspond to predicted MIR values less than 0.25 (figures 2, 3 and 5), a level of MIR below which WNV spillover has not been reported in well-studied areas of transmission (electronic supplementary material, S2.7). Coloured areas correspond to predictions which could plausibly lead to outbreaks, with higher predicted MIR values suggesting greater risk and potential size of WNV epidemics.

**Figure 4.**
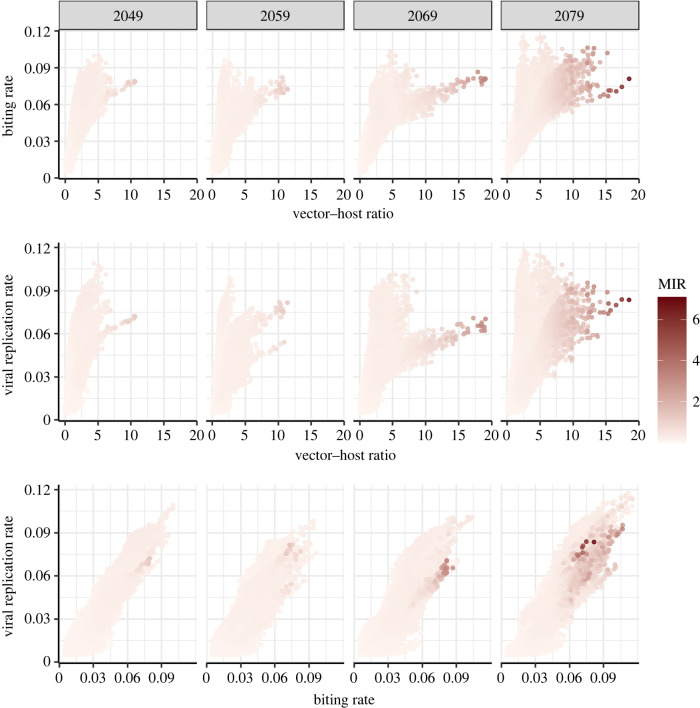
Relationships of viral replication rate, biting rate and vector–host ratio with MIR: the median viral replication rate (1/EIP), biting rate and vector–host ratio over the main vector biting season (June, July and August) are plotted with the resultant peak MIR for that season shown by the colour of the data point. The data points correspond to the simulated results across all UK grid squares and all models runs (as shown in the maps).

**Figure 5.**
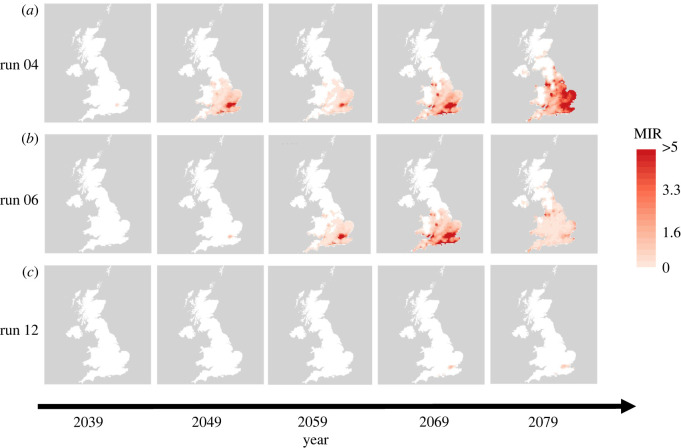
Risk of WNV outbreak under lengthened biting season: the photoperiod at which 50% of adult female mosquitoes entered diapause was decreased by 1 h, corresponding to an approximate two-week delay in diapause initiation dependent on the latitude. The plots correspond (*a*) runs 04, (*b*) 06 and (*c*) 12 and to years 2039, 2049, …, 2079.

## 3. Results

### 3.1. Epidemic risk due to migratory birds

The predicted MIR following WNV introduction via infected birds arriving at the end of March was never projected to exceed 0.25 because this was earlier than termination of mosquito diapause. Likewise, the predicted risk for late April introduction was low for all climate projections with seven of the 12 simulations in the year 2079 predicting MIR values below 0.25 across the entire UK (electronic supplementary material, SF1). In those runs for which the MIR exceeds 0.25 in some areas risks are isolated to a very small area and the predicted MIR is generally only very slightly above 0.25. Risks are predicted to be low for these earlier introduction times because we assume that adult females exiting diapause only take one blood meal before laying an egg raft and dying due to the energetic demands of diapause [30]. Transmission and amplification of WNV requires that this first spring generation has hatched and begun feeding and typically only a very small proportion of the population has met these conditions prior to the end of April when introduction takes place. If increased temperatures were also to lead to earlier exits from diapause then it is possible that predicted risks here would be higher; however, the model assumes diapause exit to be determined by increasing day length.

The predicted MIR following introduction via migratory birds at the end of May is substantially higher, as there has been sufficient time for mosquitoes to exit diapause and for the first spring generation to develop prior to arrival of the virus. Of the 12 sets of climate projections available, 11 of them resulted in predicted MIR values above 0.25 in at least one year in a least one location in the UK. In figure 2, we present the risk maps for climate runs 4, 6 and 12. These three runs were chosen to display the range of predicted scenarios, with the climate data from run 4 typically leading to relatively high predicted risk, climate run 6 leading to a moderate level of relative risk and run 12 predicting relatively low risks when comparing across all 12 sets of simulated climate data. The full set of maps for all climate runs under the late May introduction is included in electronic supplementary material, SF2. Figure 2 shows that the highest risk area is predicted to be in the southeast of England and that there is a general pattern of increasing risk through time. Predicted MIR values were below 0.25 in all areas for runs before 2039, after which point there is a general increase subject to between year variability e.g. predicted MIR in run 4 in 2059 is lower than in 2049. It is also important to note that the predicted MIR remains at low levels across the UK in approximately half the model runs, including run 12 shown. Consequently, it is perhaps unlikely, even under the worst-case scenario of RCP8.5, that WNV will become endemic in the UK and more likely that repeated outbreaks and re-introductions may occur.

A sensitivity analysis of the more uncertain parameters regarding WNV transmission between vectors and hosts is presented in electronic supplementary material, S2.8. However, one parameter which is likely to be particularly influential in the determination of WNV risk under future climate scenarios is the thermal minimum for WNV replication, *T*_min_. Due to a lack of data linking temperature to WNV replication rates in a European context, we use a laboratory-derived relationship based on a North American strain of WNV, increasing the importance of understanding the sensitivity of this parameter in particular. In figure 3, we show the estimated MIR under the same introduction scenario as in figure 2 but assuming the lower limit of the estimated 95% CI for *T*_min_ for viral replication inside the vector, which is 7.3°C (the estimated value was 11.4°C) [37]. It is clear from figure 3 that, while the qualitative patterns remain similar, the predicted WNV risk is greatly increased when the thermal minimum for WNV replication is reduced within the range of plausible values.

### 3.2. What is driving the increased risk of WNV?

The general pattern of increase in WNV risk and the prediction that outbreaks will be unlikely until the middle of the twenty-first century stems from changes to a range of processes affecting virus transmission across the mosquito biting and development season. Specifically, three of the main processes known to drive WNV transmission (hereafter also referred to as the ‘transmission processes’) are the biting rate, the vector–host ratio and the viral replication rate (the reciprocal of the extrinsic incubation period, which is the time required for the mosquito to become infectious following exposure to the virus via an infected blood meal). In figure 4, we explore the combined effects of the median values over the main mosquito biting season (taken as June, July and August) of these transmission processes on the predicted MIR across several years. We see that values in the upper end of the observed range for each of the biting rate, vector–host ratio and viral replication rate are required to facilitate WNV transmission. In other words, higher values of any individual process are not sufficient to result in a high MIR, however low values of any individual process are sufficient to inhibit transmission. Specifically, a combination of median vector–host ratios in excess of approximately five mosquitoes per bird, biting rates over around 0.06 bites per day (corresponding to a gonotrophic cycle length of approximately 17 days assuming one blood meal per cycle) and viral replication rates over 0.05 (giving an incubation time of 20 days) appear necessary for the MIR to be higher than 1, for which we might consistently expect to see outbreaks of WNV.

To understand the relative importance of each of the modelled transmission processes (biting rate, incubation period and vector–host ratio) and the interactions between these processes in determining the MIR, a series of Besag–York–Mollié 2 (BYM2) spatial models were fitted to the results of all model runs using INLA [40] (electronic supplementary material, S4). These models were evaluated from the deviance information criterion (DIC) and log score of models containing different parameter combinations [41]. Spatial models included linear and quadratic terms for each of the transmission processes and the set of models considered included all possible combinations of two-way interactions. The biting rate was transformed to its reciprocal (GC—denotes the gonotrophic cycle length, which is the time between successive blood meals) to give a linear relationship with MIR. We fit models with all main effects included and with all possible numbers and combinations of two-way interactions.

Table 1 shows the difference in DIC between the model containing all possible two-way interactions and the other models. Model 1, which includes all two-way interactions performed the best in terms of DIC and log score, implying that all interactions make some contribution to determining the predicted MIR. In comparing Models 2–4, each of which has one of the interaction terms excluded, we see that the interaction between the gonotrophic cycle and vector–host ratio is the most influential of those considered. This can be seen because Model 4, which excludes this interaction, performs poorly in comparison to the other two models, which both include it. Dropping the interaction between gonotrophic cycle duration and vector–host ratio from the best model causes the largest increase in DIC, and models with this single interaction term outperform other models with single interaction terms. In fact, the model with only the interaction between gonotrophic cycle duration and vector–host ratio outperforms the model containing both the other two interaction terms (Model 4). This finding highlights that predictions of disease can be particularly sensitive to changes in vector–host ratios and biting rates. Specifically, by comparing table 1 and figure 4 we see that the vector–host ratio appears to be the most important parameter of those considered, as the relationship between biting rate, viral replication rate and MIR is the least strong of the three relationships shown in figure 4 and the worst model in table 1 is the one which excludes all interactions involving the vector–host ratio. These findings suggest that models which make simplifying assumptions regarding vector seasonal abundance and dynamics may give poor predictions of disease risk.

**Table 1.** Table giving the log scores and ΔDIC values for a selection of the BYM2 spatial models fitted. ΔDIC values are calculated relative to the model with all three interactions included, which was found to tbe the best fitting of the models considered. Main terms for each of the vector–host ratio (VHR), gonotropic cycle (GC, reciprocal of the biting rate) and viral replication rate (VRR) are included in all models. Inclusion of a particular variable denotes inclusion of both linear and quadratic terms for that variable and inclusion of an interaction denotes inclusion of all possible interactions between both the linear and quadratic terms of those two variables e.g. linear × linear, linear × quadratic and quadratic × quadratic between the two separate variables but not linear × quadratic within a given variable.

model no.	interactions included	log score	ΔDIC
1	GC × VRR + GC × VHR + VRR × VHR	0.46	0
2	GC × VHR + VRR × VHR	0.49	−7312
3	GC × VRR + GC × VHR	0.52	−15 188
4	GC × VRR + VRR × VHR	0.60	−34 389
5	GC × VHR	0.58	−29 974
6	VRR × VHR	0.68	−55 104
7	GC × VRR	0.91	−11 4104
8	—	1.01	−14 1039

### 3.3. Effects of lengthened biting season

The model used here is parameterized such that 50% of adult female mosquitoes will enter diapause by a photoperiod of 15 h per day, which coincides with early-to-mid-August in the UK [30,36]. However, diapause initiation is known to be dependent on both photoperiod and temperature [36]. Furthermore, mosquitoes in the Mediterranean basin are known to continue biting in high numbers throughout August and into September [42], and indeed most WNV cases in Europe occur in the autumn [26]. Consequently, it is thought that increased temperatures may also result in mosquitoes in the UK remaining active later into the season [43]. Figure 5 shows that if the mosquito season were to increase by approximately two weeks then the predicted risk would increase substantially across all climate realizations (cf. figure 2). We now predict substantial risk as early as the middle of the century, though risks of outbreaks in 2039 and earlier remain low. This substantial increase in risk following a lengthening of the biting season stems from the consequent increase in the amount of time over which virus amplification can occur and from the increase in vector density due to the longer active season and shorter diapause period.

## 4. Discussion

We predict that, while current UK temperatures appear too low for WNV transmission cycles to be established, projected increases to UK temperatures in the coming years will increase the risks of WNV outbreaks, with epidemics appearing possible by the second half of the century (figure 2). The outbreak risk is predicted to increase as WNV introduction occurs later in the period from March to May and the risk is highest in southeast England. However, the rate at which risk increases over time is strongly dependent on the relationship between WNV replication rates and temperature in the vector, which is not currently well understood across the range of flaviviruses potentially circulated by European mosquito populations [44]. The degree to which increased temperatures may increase the length of the mosquito biting season is also predicted to have a large impact on outbreak risk, with longer seasons leading to substantial increases in both the area at risk and the size of outbreaks (figure 5).

By explicitly modelling the seasonality in the vector population, our framework allows us to investigate the risks associated with pathogen introduction at different times of the year. The ability to capture the effects of this synchronicity between vector seasonality and host dispersal is important in estimating establishment risk across several VBDs, including tick-borne diseases for which bird migration has been implicated in the spread of ticks and tick-borne pathogens across Europe [45]. We have shown the predicted MIR in the mosquito population following introduction of WNV at a range of locations in the UK and highlighted that only infected birds late in the migration window were likely to cause outbreaks given current assumptions regarding diapause. Our prediction that the highest risk area for establishment of WNV cycles is in the southeast coincides with the findings of Bessell *et al.* [27]. Areas of the southeast have also recently become home to populations of *Cx. modestus*, which acts as a bridge vector between avian and human hosts [46]. This combination of high predicted transmissibility, a suitable migratory bird population and a known bridge vector highlight that an area such as the South Kent marshes should be a priority for any WNV surveillance in the UK. This is particularly true since changes to WNV transmission in France, which Bessell *et al.* [27] considered as the potential source of infected migratory birds, and general northward expansion of WNV in Europe [23] will likely continue to increase the introduction risk of WNV in the coming years.

The framework presented is a valuable tool by which to assess the potential effects of long term, climate-driven changes in VBDs. Despite widespread predictions that increased temperatures will increase vector biting seasons and potential windows for pathogen transmission, there is little empirical evidence to quantify these effects to date, potentially due to the large-scale, long-term monitoring which would be required [47]. Indeed, even if these effects could be quantified in endemic regions, mechanistic modelling approaches would still be required to provide predictions in potential epidemic zones [48]. Studies into the seasonality of *Cx. pipiens* in the UK are limited, however existing predictions suggest that the biting season continues until approximately mid-August [30]. However, in central Europe and the Mediterranean basin, where temperatures are generally warmer and seasonal variation in photoperiod is less, *Cx. pipiens* are known to continue biting throughout the month of August and into September [42]. Previous models have predicted that climate change will increase the biting season of *Cx. pipiens* and consequently the transmission season and geographical distribution of WNV [49,50]. We have shown that an increase to the length of the mosquito biting season of even two weeks has profound increases to the extent of WNV transmission predicted, principally due to the increase in vectors per host.

By developing a mechanistic model which captures the vector life cycle and the interaction between the vector and host populations we are able not only to investigate potential disease risk but also to understand the relative importance of different aspects of the vector life cycle on transmission. We have shown that high biting rates and viral replication rates do not necessarily lead to high predicted infection rates if the number of vectors per host is not also sufficiently high (figure 4). This suggests that perhaps shorter periods of extreme warm weather, which are predicted to become more common in coming years [32], may not lead to substantially increased WNV risk if they only serve to increase biting rates and viral replication rates for a time. Rather, it may be cumulative effects of increased temperatures over whole seasons, perhaps coupled with longer periods of weeks or months of extreme temperatures, which lead to larger vector populations and consequently increased risk of VBD. While known to be an important determinant of disease risk, the vector–host ratio is also one of the most difficult parameters to estimate in VBD models [22,51] due to large geographical variability and the requirement of extensive field data. Consequently, we propose that accurate estimation of vector–host ratios should be a priority when estimating establishment of VBDs.

The model also highlights the importance of understanding the precise relationship between temperature and pathogen replication rates at a range of temperatures and how these might vary geographically across different vector populations or different strains of pathogens (figures 2 and 3). However, for many VBDs, such as WNV, these relationships are not widely studied and rely on extrapolation from a small number of studied vector–pathogen combinations, which may not be representative of the population in question [37,52]. Our modelling framework provides a valuable tool by which a range of hypothetical scenarios can be investigated and allows identification of situations in which further laboratory studies are required to improve predictions of disease risk, as would be beneficial in the case of WNV in the UK.

We have made the simplifying assumption in this work that there is no spatial variability in the extent of vector breeding habitat. This is clearly untrue, however translating the effect of changing rainfall, a known predictor of habitat [42], into a measure of available breeding habitat and coupling this habitat availability with disease transmission presents a wide range of challenges. These challenges include the variability in types of breeding habitat used across, and even within, vector species and the complex relationships between habitat availability and disease incidence [53]. For example, *Cx. pipiens* use a wide range of containers for breeding, ranging from small transient sites (e.g. cow hoofprints) to larger more permanent sites (e.g. marshes, ponds and water butts) [54]. Human behaviour is thought to affect site availability as people store water in times of low rainfall, creating artificial habitats. Furthermore, despite a detrimental effect on natural habitat availability, periods of drought have been proposed as the main climatic driver of WNV in the USA, where WNV has invaded [55], as they lead to increased host–vector contact, which increases infection prevalence in mosquitoes [56]. Indeed, changing rainfall patterns and potential extreme rainfall events are expected to affect a wide range of vectors in conflicting ways, for example through changes to mosquito habitat availability, flushing of mosquito larvae during period of extreme rainfall, or through soil moisture effects on tick development and survival [57]. Given the complex links between hydrology, host–vector dynamics and disease spillover into humans we have focused on the effects of temperature in this study. However, it is likely that this simplification may result in underestimates of vector–host ratios in ideal habitats and overestimates in others. Consequently, exploration of approaches by which hydrology can be integrated into VBD models using the increasing amounts of remotely sensed climate data available, as has recently been done for schistosomiasis and trypanosomiasis [58], should be a priority for further research.

We highlight that our modelling framework, combining environmentally forced stage-structured DDEs capturing the vector life cycle with equations representing host and pathogen dynamics, could be applied to study establishment risk of a wide range of VBDs across temperate regions under projected climate scenarios. While the model presented here focusses specifically on WNV, modification of the pathogen replication rate would readily allow other mosquito-borne diseases of risk in Europe, such as Usutu, to be studied [59] and small modifications to the structure or parameterization of the host equations would allow the framework to be applied to diseases of concern such as Rift Valley fever [60].

The model presented predicts that WNV outbreaks in the UK are unlikely given current temperatures, though the outbreak risk is predicted to increase as temperatures warm throughout the century. Nonetheless, these predictions are based upon temperatures simulated under RCP8.5, which is intended to describe a worst-case scenario with regards to continued investment in fossil fuels and no effort to reduce greenhouse gas emissions [61]. Furthermore, only some of the realizations from this climate model lead to appreciable levels of risk towards the second half of the century. Consequently, our findings suggest that if efforts to limit greenhouse gas emissions and keep warming at low levels are successful then WNV outbreaks in the UK could at least be mitigated against, if not avoided completely, depending on how vector active season lengths are influenced by warming temperatures.

## Data accessibility

All code is available on GitHub at [38].
